# Ultrafiltration versus Diuretics on Prognostic Cardiac and Renal Biomarkers in Acute Decompensated Heart Failure: A Systematic Review and Meta-Analysis

**DOI:** 10.3390/jcm12082793

**Published:** 2023-04-09

**Authors:** Kirsty Luo-Yng Tay, Abdel Rahman Osman, Esyn Ee Xin Yeoh, Jasmine Luangboriboon, Jie Fei Lau, Joanne Jia An Chan, Majed Yousif, Benjamin Yi Hong Tse, Graham Horgan, David T. Gamble, Phyo Kyaw Myint

**Affiliations:** 1 School of Medicine, Medical Sciences and Nutrition, University of Aberdeen, Aberdeen AB25 2ZD, UK; 2 Biomathematics & Statistics Scotland, Aberdeen AB25 2ZD, UK; 3 Ageing Clinical & Experimental Research (ACER) Team, Institute of Applied Health Sciences, University of Aberdeen, Aberdeen AB25 2ZD, UK; 4 Aberdeen Cardiovascular & Diabetes Centre (ACDC), Institute of Medical Sciences, University of Aberdeen, Aberdeen AB25 2ZD, UK

**Keywords:** ultrafiltration, diuretics, heart failure, creatinine, brain natriuretic peptide, sodium

## Abstract

Existing systematic reviews have insufficiently delineated the differing cardiac and renal profile of ultrafiltration compared to diuretics as a method of decongestion in acute decompensated heart failure. This meta-analysis will investigate the impact of ultrafiltration compared to diuretics on prognostic cardiac and renal biomarkers. We searched PubMed Central, Ovid MEDLINE^®^, Ovid Embase, all EBM reviews, and Web of Science Core Collection for randomised controlled trials published before 21 July 2022. Our main outcome measures were cardiac (brain natriuretic peptide and N-terminal pro-brain natriuretic peptide) and renal biomarkers (serum creatinine, serum sodium, and blood urea nitrogen). A total of 10 randomised trials were included in our analysis after screening. An inverse-variance random effects meta-analysis of the pooled results demonstrated no significant difference between ultrafiltration and diuretics for brain natriuretic peptide, N-terminal pro-brain natriuretic peptide, creatinine, sodium and long-term blood urea nitrogen. However, ultrafiltration produced statistically greater increases in blood urea nitrogen in the short-term (mean difference, 3.88; 95% confidence interval 0.59–7.17 mg/dL). Overall, ultrafiltration produces a similar impact on prognostic cardiac and renal biomarkers when compared to diuretic therapy. We highlight ultrafiltration’s significant impact on short-term BUN and recommend further research to investigate more optimal protocols of ultrafiltration administration.

## 1. Introduction

Acute decompensated heart failure (ADHF) represents 50–70% of all acute heart failure presentations. It is characterised by systolic dysfunction and fluid overload in the context of pre-existing heart failure or cardiac dysfunction [1]. The resultant fluid overload is predominantly managed by loop diuretics such as furosemide. Although effective, prolonged loop diuretic usage risks significant neurohormonal activation and diuretic resistance [2].

Ultrafiltration has garnered interest as an alternative to diuretics after multiple randomised clinical trials demonstrated greater fluid removal and reduced neurohormonal activation [3,4,5,6,7]. While its adoption within clinical practice has been limited, prior meta-analyses have showcased its advantage in rehospitalisation rates (absolute risk reduction of 10.9% at longest follow-up) and fluid removal (decrease in mean body weight of −1.8 kg; 95% CI −4.68 to 0.97 kg at longest follow-up) [8,9]. The efficacy of stepped pharmacological care, as demonstrated by CARRESS-HF, contests the role of this expensive therapy with a less familiar safety profile [10].

Ultrafiltration provides neurohormonal advantages with regards to reduced renin-angiotensin-aldosterone system (RAAS) activation and superior natriuresis. Superior diuresis potentially improves cardiac function by reducing ventricular stretching, resulting in a decrease in brain natriuretic peptide (BNP) and N-terminal pro-brain natriuretic peptide (NT-proBNP). A decrease in either is associated with a lower incidence of adverse events when followed up at 12 months [11]. With many studies limiting follow-up to 90 days, the inclusion of these prognostic biomarkers allows for the prediction of longer-term differences in prognosis [12].

The impact of this superior decongestion on renal and biochemical prognostic markers is only partially delineated, with prior systematic reviews including only serum creatinine and eGFR. However, ultrafiltration’s renal profile is multifaceted, with increased natriuresis and diuresis producing an unknown impact on the markers of renal hypoperfusion (Blood Urea Nitrogen, BUN) and electrolyte stability (serum sodium). Both markers carry significant prognostic power, with elevated BUN and hyponatraemia contributing to increased morbidity and mortality [13].

Against this background, we aim to systematically review and synthesise the available evidence on the effects of ultrafiltration versus diuretics on BNP, NT-proBNP, serum sodium, serum creatinine and BUN. In the remainder of this meta-analysis, the terms “sodium” and “creatinine” refer exclusively to “serum sodium” and “serum creatinine” respectively. 

## 2. Materials and Methods

The protocol for this meta-analysis is registered with Prospero (CRD42022347112). We searched PubMed Central, Ovid MEDLINE^®^ (1946 to 21 July 2022), Ovid Embase (1947 to 21 July 2022), all EBM reviews and Web of Science Core Collection on 21 July 2022. The free search terms were: (heart failure or cardiac failure) AND (ultrafiltration or mechanical fluid removal or haemodialysis or aquapheresis) AND (diuretics or diuretic agent or loop diuretics or thiazide diuretics or water pill).

The Population, Intervention, Comparator, Outcome framework was used to formulate our eligibility criteria [14]. Only non-substudy randomised controlled trials (RCTs) with published results (including interim) were included. Included RCTs recruited patients aged 18 and older whose primary diagnosis was ADHF. To be included, RCTs must have reported at least one of BNP, NT-proBNP, BUN, creatinine and sodium. RCTs that included diuretic resistant patients were excluded.

**Figure 1:**
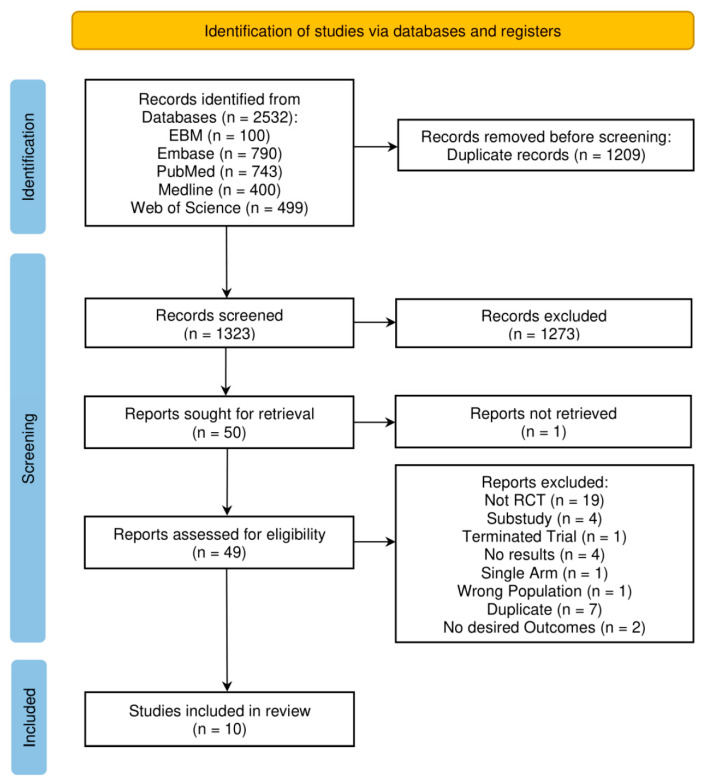
PRISMA flow chart representing the literature screening process [15].

Titles and abstracts were independently deduplicated and screened by four authors based on our eligibility criteria. Consensus was met regarding the final included studies amongst all authors. Our screening methodology is detailed in the Preferred Reporting Items of Systematic reviews and Meta-Analyses (PRISMA) diagram [15] (Figure 1). Study characteristics, protocols, and outcome data were doubly extracted by six independent reviewers. To allow for cross-comparison between studies, units were standardised. Non-physiological creatinine data reported by Hanna et al., 2012 in μmol/L was corrected to mg/dL [6]. Marenzi, 2014 provided values for urea that were converted to BUN and included in our meta-analysis [16]. 

Each study was independently appraised at least twice using the Critical Appraisal Skills Programme (CASP) RCT Standard Checklist, with discrepancies resolved through discussion [17]. Four reviewers independently assessed risk of bias using the revised Cochrane risk of bias tool for randomized trials [18]. Bias assessment for creatinine was considered a surrogate assessment for BUN and sodium, as BNP was for NT-proBNP. Discrepancies were resolved through discussion.

Microsoft Excel (Microsoft Corporation: Redmond, WA, USA) was used to visualise pooled outcome data for each biomarker against Log_10_ (time). This demonstrated the effect of treatment duration on the mean difference between ultrafiltration and diuretics, hence determining the suitability of comparing data across different time-points (Supplementary Materials, Figures S1–S5). Only BUN showed a difference in trend between short-term (before discharge) and long-term (after discharge) follow-up. 

Revman Version 5.4 (Cochrane: London, UK) was used to perform an inverse-variance, random effects (to account for heterogeneity) meta-analysis and I² statistical analysis of heterogeneity for each biomarker. Data from the latest time point were used with three exceptions. In these three cases, we used data for BNP, BUN, and sodium reported by Marenzi, 2014 at discharge, as opposed to 12 months, to allow for imputation of a standard deviation (SD) for change. Short-term (before discharge) and long-term (after discharge) BUN were analysed separately. We converted data for all outcomes into ‘change from baseline’ values. For sodium, we used magnitude of change from baseline since smaller changes, regardless of direction, indicate a superior safety profile. Missing SDs for change were obtained either through contacting study authors, only successful in the case of Bart, 2005, or imputation. SDs were imputed from studies reporting SDs at a similar time point, with a mean SD calculated when multiple imputations were possible. In the case of Şeker, 2016, SDs for change in serum creatinine were calculated from the p value between arms. Sensitivity analysis was performed for outcome measures when I² was greater than 50%, SDs were imputed, or if the latest time point was not used [19].

## 3. Results

The study selection process is summarised in Figure 1. Our search strategy produced 2532 articles, of which 1209 were duplicates. Of the remaining 1323 publications, 1273 were excluded through title and abstract screening. The remaining 50 publications underwent full-text screening, and 10 were selected for inclusion in this review [3,4,5,6,7,10,16,20,21,22].

Critical appraisal using the CASP checklist demonstrated that specific randomisation methods and allocation concealment were unspecified in six papers. None of the study interventions were blinded to participants and investigators. The precision of the treatment effect (confidence interval) was reported by four studies.

A summary of study characteristics is shown in Table 1. The 10 included RCTs reported data from Canada, Italy, Turkey, China and the USA. This corresponds to a total of 917 participants, with 443 and 474 participants randomised to ultrafiltration and diuretics, respectively. The sample size of participants across all studies ranged from 16 to 221. The mean participant age was 67 years, with 71.3% of participants being male.

A summary of study protocols is depicted in Table 2. Various devices, including Aquadex 100 (CHF Solutions, Inc.; Brooklyn Park, MN, USA), PRISMA system (Baxter International Inc.; Deerfield, IL, USA), NxStage system One (NxStage Medical, Inc.; Lawrence, MA, USA), Aquadex FlexFlow System (CHF Solutions, Inc.; Brooklyn Park, MN, USA), and FQ-16 type ultrafiltration dehydration device (Beijing Hartcare Medical Technology Co., Ltd.; Beijing, China), were used to administer ultrafiltration therapy at a flow rate ranging from 100 mL/h to 500 mL/h. Several discrepancies existed between study protocols with regard to number of ultrafiltration sessions, provision of vasoactive medications, and loop diuretic use in the interventional arm.

**Figure 2:**
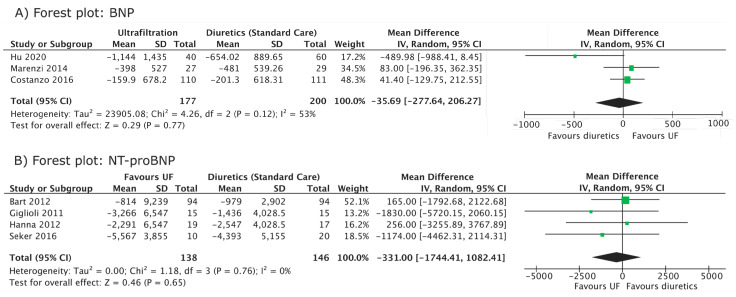
Forest plot of cardiac biomarkers (BNP and NT-proBNP). (**A**) Forest plot comparing the mean difference in BNP (change from baseline) between ultrafiltration and diuretic groups. (**B**) Forest plot comparing the mean difference in NT-proBNP (change from baseline) between ultrafiltration and diuretic groups. Individual studies are represented by their author and year of publication in the first table column. Mean differences and 95% confidence intervals are presented for individual studies. IV, inverse variance; CI, confidence interval [4,5,6,10,16,17,22].

BNP was followed up by three studies [5,16,21] (Figure 2A, Table 3). There was no significant difference between diuretic and ultrafiltration arms (mean difference, −35.69 pg/mL; 95% CI −277.64, 206.27, I^2^ = 53%). A sensitivity analysis, in which each study was excluded in turn, did not affect the significance of the overall results.

NT-proBNP was followed up by four studies [4,6,10,22] (Figure 2B, Table 3). No significant difference was demonstrated between the diuretic and ultrafiltration groups (mean difference, −331 pg/mL; 95% CI, −1744.41, 1082.41, I^2^ = 0%). The individual and combined exclusion of Giglioli 2011 and Hanna 2012, in which SDs were obtained through imputation, did not affect the significance of the overall results.

**Figure 3:**
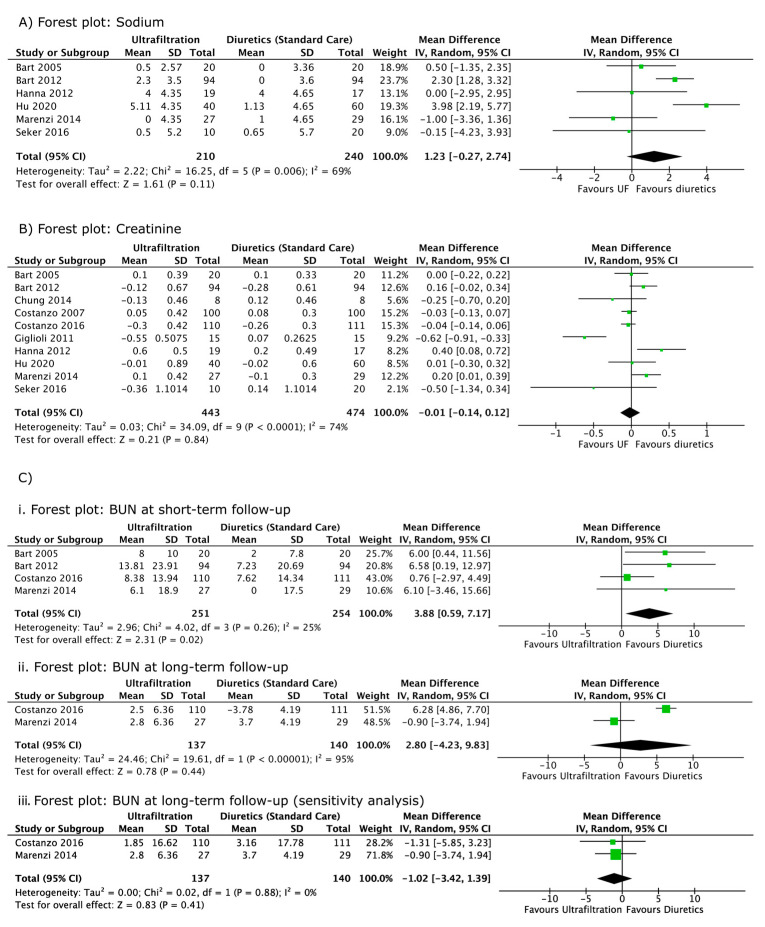
Forest plot of renal biomarkers (sodium, creatinine and BUN). (**A**) Forest plot demonstrating the mean difference in sodium (change from baseline) between ultrafiltration and diuretic groups. (**B**) Forest plot comparing the mean difference in creatinine (change from baseline) between ultrafiltration and diuretic groups. (**C**) (**i**) Forest plot comparing mean difference in short-term BUN (only includes data reported before or at discharge) between ultrafiltration and diuretic groups. (**ii**) Forest plot comparing the mean difference in long-term BUN (only includes data reported after discharge) between ultrafiltration and diuretic groups. Heterogeneity demonstrated by the I_2_ test is 95%. (**iii**) Forest plot demonstrates the impact of a sensitivity analysis (using data reported by Costanzo, 2016 at 2 instead of 3 month) on mean differences in long-term BUN. Standard deviations for Marenzi, 2014 at 12 months were imputed from Costanzo, 2016 at 3 month. Individual studies are represented by their author and year of publication in the first table column. Mean differences and 95% confidence intervals are presented for individual studies. IV, inverse variance; CI, confidence interval [3,4,5,6,7,10,16,17,21,22].

Serum sodium was followed up in six studies [3,6,10,16,21,22] (Figure 3A, Table 3). The pooled results suggest no significant difference in the magnitude of change of sodium between the ultrafiltration and diuretic arms (mean difference, 1.23 mmol/L; 95% CI, −0.27, 2.74, I^2^ = 69%). A sensitivity analysis that was performed with a combined exclusion of the imputed results from Hanna, 2012, Marenzi, 2014, and Hu, 2020 did not change the significance of the overall results but reduced heterogeneity (I^2^ = 46%).

Serum creatinine was followed up in 10 studies [3,4,5,6,7,10,16,20,21,22] (Figure 3B) (Table 3). The pooled results suggest no significant difference between the diuretic and ultrafiltration arms (mean difference, −0.01 mg/dL; 95% CI, −0.14, 0.12, I^2^ = 74%). Individual and grouped sensitivity analyses that were performed on studies with imputed SDs had no influence on the significance of the overall results. Grouped exclusion of these studies lowered I^2^ to 34%, while the individual exclusion of Giglioli 2011 reduced I^2^ to 51% [4,6,7,15,20].

BUN or urea was followed up in five studies [3,5,7,10,16] (Figure 3C) (Table 3). Costanzo 2007 [7] reported non-significant changes without numerical values and was therefore excluded from our analysis. In the short-term, diuretics produced a significantly smaller increase in BUN (mean difference, 3.88 mg/dL; 95% CI 0.59, 7.17, I^2^ = 25%) as compared to ultrafiltration (Figure 3Ci). No significant difference was observed between the interventional and the control arms in the long term (mean difference, 2.80 mg/dL; 95% CI, −4.23, 9.83, I^2^ = 95%) (Figure 3Cii). The use of data for Costanzo, 2016 [5] reported at two instead of three months reduced heterogeneity from 95% to 0%, without affecting the significance of the analysis (Figure 3Ciii).

The risk of bias assessment of BNP showed some concerns in all three studies that reported this outcome (Supplementary Materials, Figures S6 and S7). This was primarily due to a lack of specification of the mode of analysis pre-randomisation, resulting in potential bias in the selection of the reported result. Regarding serum creatinine, seven studies displayed some concerns [3,5,6,10,16,20,21], and three studies displayed a high risk of bias. Costanzo, 2007 [7], Giglioli 2011 [4], and Şeker, 2016 [22] all yielded a high risk of bias due to either a lack of intention to treat analysis or an absence of an alternative method to account for dropouts (Supplementary Materials, Figures S8 and S9).

## 4. Discussion

Our meta-analysis demonstrated that ultrafiltration similarly impacts markers of cardiac and renal function in ADHF as compared to diuretics. Ultrafiltration and diuretics displayed similar improvements in reducing cardiac biomarkers (BNP and NT-proBNP). Both therapies have a similar impact on renal biomarkers, with no significant difference between arms for creatinine, sodium and (long-term) BUN. Ultrafiltration demonstrated a slight inferiority with regards to BUN in the short term (Figure 3).

The most recent meta-analysis by Srivastava et al., 2022, which focused on adverse events and markers of decongestion, concluded that ultrafiltration significantly reduced long-term rehospitalization, whilst preserving renal function [8]. However, it is worth noting their inclusion of a study by Tabak’ian, et al., which utilised haemofiltration as opposed to ultrafiltration. Their meta-analysis on creatinine omitted two of their included studies [7,22] and used imputed SDs from one study [23]. Heterogenous follow-up durations justified these imputations in all but three instances where SDs were available in a manuscript or trial registry. Our updated analysis demonstrates that these methodological differences do not affect our common conclusion: ultrafiltration’s effect on creatinine is statistically similar to diuretics.

As demonstrated by UNLOAD [7] and AVOID-HF [5], ultrafiltration is associated with greater volumes of fluid removal alongside lower rates of rehospitalisation without compromising renal function. Although these findings were broadly replicated [3,4,6], CARRESS-HF [10] concluded that while ultrafiltration therapy maintained similar levels of decongestion, it produced significantly worse renal function in the short and long term. While UNLOAD and AVOID-HF consider the use of ultrafiltration before renal deterioration, CARRESS-HF only includes patients with worsening renal function. Though this might suggest that patients with deranged renal function are poor candidates for ultrafiltration therapy, it is more convincing that the use of fixed-rate ultrafiltration (unguided by renal status) negatively impacted the renal outcomes of patients receiving ultrafiltration as compared to an adjustable diuretic regime [5]. CARRESS-HF also reports a significantly greater reduction in sodium (*p* < 0.001). An analysis of CARRESS-HF by Kitai et al., 2017 showed that while treatment-induced (short-term) hyponatraemia was not associated with worse clinical outcomes, discharge (persistent) hyponatraemia was associated with increased all-cause deaths, re-hospitalisations or unscheduled hospital visits. Persistent hyponatraemia was significantly more common within the ultrafiltration arm [24]. This disparity may be attributed to the fixed ultrafiltration regime within the CARRESS-HF protocol, especially as this was not replicated in studies that allowed greater physician discretion [3,6,16,21,22].

Existing systematic reviews have concluded that ultrafiltration has a benign renal profile without including BUN within their outcomes [8,9,25,26,27]. Although BUN was measured in five studies [3,5,7,10,16], it has not been sufficiently discussed within RCT manuscripts. While CARRESS-HF reports a significantly greater increase in BUN at 96 h in the ultrafiltration arm, the relevance of these results were not discussed. Our meta-analysis confirms that ultrafiltration likely leads to greater increases in BUN in the short-term, but it does not lead to statistically significant difference in long-term BUN (Figure 3). We propose that intravascular depletion, a potential consequence of ultrafiltration, may result in short-term increases in BUN through renal hypoperfusion and increased urea reabsorption at the nephron [28,29]. The reversal of this transient depletion with time likely explains why our meta-analysis showed non-significant differences in BUN between arms in the long-term [3]. As our longer-term analysis only included two studies [5,16], future studies should follow-up BUN in the long term to confirm the nature of this trend.

Ultrafiltration’s decongestive superiority might be attributed to a reduced stimulation of the macula densa and RAAS activation, resulting in more isotonic fluid removal [16]. While this decongestive superiority might be expected to translate to less ventricular stress and hence lower levels of BNP/NT-proBNP, this was not reflected in five of our included studies [5,6,10,16,22]. Contrastingly, two papers [4,21] found that ultrafiltration was significantly more effective at reducing BNP/NT-proBNP compared to diuretics. In the study published by Hu 2020 [21], this may be explained by the sequential administration of ultrafiltration along with diuretics, which has the potential to reverse the braking phenomenon brought on by chronic diuretic administration. In the braking phenomenon, gradually diminishing natriuresis due to increased sodium reabsorption in the distal nephron results in diuretic resistance. This increased sodium reabsorption may be attributed to distal tubular remodelling worsened by heightened RAAS activation and hypochloraemia [30,31]. Hypochloraemia, due to loop diuretics, has been shown to increase WKN1 and WKN4 activity and hence stimulate both Na-K-Cl and Na-Cl cotransporters. The addition of another diuretic, for the purpose of sequential nephron blockade, allows for simultaneous diuretic activity at multiple nephron sites [31]. The CARRESS-HF trial postulates the decongestive equivalency of this method as compared to ultrafiltration in patients with ADHF and cardiorenal syndrome. However, flexible sole ultrafiltration therapy given before the development of renal impairment is still likely superior to stepped diuretic therapy in reducing fluid overload [5].

Modulating ultrafiltration treatment parameters may further increase the superiority gap. A meta-analysis by Wang et al., 2021 recommends mean fluid removal to be set at ≥ 200 mL/h after their subgroup analysis demonstrated superior fluid removal, weight loss, and rehospitalisation rates [27]. We hypothesize that flow rate, which differs substantially between studies, may play a similar role in affecting cardiac and renal biomarkers. The absence of a subgroup analysis (due to insufficient reporting) caveats the certainty of our conclusion at different flow rates. We recommend that future research executes a subgroup analysis with an analysis of covariance (ANCOVA) on individual patient data to delineate the nature of this relationship. Future studies could also consider a dual interventional approach at higher and lower flow rates to investigate this hypothesis.

The use of diuretics within the ultrafiltration arm of both Bart et al., 2005 [3] and Marenzi et al., 2014 [16] may impact the estimation of the differential effect size of ultrafiltration as compared to diuretics. Without an ANCOVA, the effect of diuretics and other additional medical therapies cannot be accurately determined. While differences in dosage were not statistically significant between arms in either study [3,16], significantly less patients in the ultrafiltration arm of Bart, 2005 were given diuretics (*p* = 0.044). Even if the effects of diuretics and ultrafiltration within the interventional arm are independent of each other (i.e., there is no synergistic/antagonistic effect), the uneven use of diuretics between arms implies that the mean difference is not an accurate representation of sole ultrafiltration therapy. However, as heart failure patients often present acutely unwell, sole ultrafiltration administration might be insufficient. Hence, we acknowledge that whilst this is a limitation of the study protocol, it is not out of the trial context.

The large variability in ultrafiltration (flow rate and duration) and diuretic treatment protocols reflecting real-world uncertainty is a limitation of our meta-analysis. The substantial I² heterogeneity of BNP and creatinine suggests that treatment protocol and/or follow-up time inconsistencies may produce divergent outcomes. Our meta-analysis is further hindered by the incomplete reporting of study protocols and individual patient data that prevents a sub-group analysis or ANCOVA. By design, our meta-analysis is focused and limited in scope. We do not re-address ultrafiltration’s impact on morbidity, mortality, or decongestive outcomes, as this has been covered extensively in the literature [8,9,25,26,27].

Ultrafiltration followed by sequential diuretics is a potential strategy that may further alleviate fluid overload and reverse diuretic resistance without compromising renal function [21]. Further research in the form of trials comparing the sequential administration of ultrafiltration and diuretics (ultrafiltration followed by diuretics versus diuretics followed by ultrafiltration) is needed to determine the potentially temporal synergism of co-administration. To allow for clinically relevant conclusions, we recommend that future studies adopt a stepped pharmacological protocol within their diuretic arm. Studies should also consider investigating the effects of ultrafiltration flow-rate to determine if greater decongestion at higher flow-rates is associated with a changed cardio-renal picture. Improvements in the consistency of reporting (follow-up times and protocol) should also be attempted by future RCTs to enhance the accuracy of meta-analyses.

## 5. Conclusions

In summary, while ultrafiltration presents a stable but unique renal profile, transient intravascular depletion may result in an initial divergence from the expected clinical norms of diuretic therapy. Clinicians should note that a flexible ultrafiltration rate guided by the clinical and biochemical picture is likely to avoid adverse renal outcomes and decrease the risk of persistent hyponatraemia. Ultrafiltration appears to be most beneficial when given earlier (before renal deterioration), as this strategy is not associated with poor renal outcomes. While ultrafiltration is no better at improving cardiac biomarkers when used alone, it maintains decongestive superiority over diuretics and is potentially superior in improving cardiac biomarkers when used in combination with diuretics.

## Figures and Tables

**Table 1:** Characteristics of population and study type for included studies.

	Bart [3]	Costanzo [7]	Giglioli [4]	Bart [10]	Hanna [6]	Chung [21]	Marenzi [17]	Costanzo [5]	Şeker [22]	Hu [16]
Year	2005	2007	2011	2012	2012	2014	2014	2016	2016	2020
Trial	RAPID-CHF	UNLOAD	ULTRADISCO	CARRESS-HF	-	-	CUORE	AVOID-HF	-	-
Country	USA	USA	Italy	Canada, USA	USA	USA	Italy	USA	Turkey	China
Sample size	40	200	30	188	36	16	56	221	30	100
Intervention (UF) cohort size	20	100	15	94	19	8	27	110	10	40
Diuretics cohort size	20	100	15	94	17	8	29	111	20	60
Mean ± SD age of UF cohort	67.5 *	62 ± 15	72.4 ± 14.1	68.9 ± 12.0	60 ± 9.1	69 ± 14	75 ± 8	67 ± 13	66.5 ± 9.8	70.6 ± 10.44
Mean ± SD age of diuretics cohort	69.5 *	63 ± 14	65.8 ± 18.4	67.1 ± 13.7	59 ± 15.5	74 ± 12	73 ± 9	67 ± 13	66.8 ±10.2	73.52 ± 9.83
% Male in UF	70	70	87	78	84.2	87.5	81	69.1	60	55
% Male in diuretics	70	68	87	72	76.5	100	83	73	65	55

UF, ultrafiltration; % percentage; * represents median values.

**Table 2:** Summary of study protocols.

Study ID	Protocol for Ultrafiltration (UF) Group	Protocol for Diuretics Group
Bart et al., 2005 (RAPID-CHF) [3]	System 100 was used for a single 8 h course with fluid removal rates determined by the attending physician (up to a maximum of 500 cc/h). For the duration of UF, diuretics were withheld. Additional courses of UF were allowed at the discretion of treating physicians after 24 h endpoints were assessed. The median cumulative dose of furosemide received during the first 24 h was 80 mg. The median volume of ultrafiltrate removed was 3213 mL.	Standard therapies as per local guidelines were given. The median cumulative dose of furosemide received during the first 24 h was 160 mg.
Costanzo et al., 2007 (UNLOAD trial) [7]	Aquadex System 100 was used with a blood flow ranging between 10 and 40 mL/min and with a total blood volume of 33 mL. The duration and rate (up to 500 mL/h) were decided by the treating physician. Intravenous diuretics were prohibited in the first 48 h after enrolment. Administration of IV vasoactive drugs were not prohibited, but patients requiring it 48 h post-randomisation were considered to have failed treatment. The mean rate of fluid removal was 241 mL/h for 12.3 ± 12 h.	Intravenous diuretics were used. The dose of diuretics had to be at least twice the dose of pre-hospitalisation. A total of 68 patients received bolus injections, and 32 received continuous infusion of intravenous diuretics. The mean dose of diuretics given daily was 181 ± 121 mg during the 48 h after randomisation.
Giglioli et al., 2011 (ULTRADISCO) [4]	PRISMA System was used (M 100 PRESET PRISMA filter) with an blood flow rate of 150 mL/h. Continuous UF technique was used, with the rate of fluid removal ranging from 100 to 300 mL/h, which was adjusted according to the response. The duration of UF differed according to the clinical condition of the patient. Intravenous diuretic therapy was discontinued during UF treatment. Administration of IV inotropes were not prohibited, but patients requiring it post-randomisation were considered to have failed treatment (no patients required inotropes).	Continuous infusion of furosemide at an initial dose of 250 mg/24 h. This was reduced or increased, depending on patient response. The maximum dose was 500 mg/24 h. Administration of IV inotropes were not prohibited, but patients requiring it post-randomisation were considered to have failed treatment (no patients required inotropes).
Bart et al., 2012 (CARRESS-HF) [10]	The Aquadex System was used with a fluid removal rate of 200 mL/h. Loop diuretics were withheld for the duration of UF treatment in the intervention group. Additional treatments (with vasodilators or positive inotropes) were discontinued after randomisation unless deemed necessary as life-saving therapies.	Intravenous diuretics were used, and the dose was adjusted to maintain a urinary output of 3 to 5 L/24 h. Treatment was continued by the treating physician until volume status (based on blood pressure, physical exam findings, haemodynamics, BUN, and creatinine) was optimised. The use of IV vasodilators and inotropic agents was allowed in patients that did not meet their target urine output.
Hanna et al., 2012 [6]	NxStage System One was used with a blood flow rate of 200 to 300 mL/min. UF rate was set at 400 mL/h for 6 h and then decreased to 200 mL/h (changes were permitted if clinically indicated). All diuretics except for spironolactone (≤25 mg/dL) were stopped during UF treatment. Intravenous vasoactive medication was used under the discretion of the treating physician based on haemodynamic targets. Vasoactive doses were only reduced and never increased.	IV diuretics were used at doses and frequencies designated by the treating clinician. Intravenous vasoactive medication was used under the discretion of the treating physician based on haemodynamic targets.
Chung et al., 2014 [21]	The Aquadex 100 system was to achieve a target weight removal that was established by the heart failure service. The mean UF rate was 162 mL/h. Loop diuretics were discontinued in patients in the UF group after randomisation.	Continuous intravenous furosemide infusions were given to achieve the removal of a target weight established by the heart failure service.The mean daily dose of furosemide was 212 mg.
Marenzi et al., 2014 (CUORE Trial) [17]	A simplified Device (Peristaltic pump and polysulphone filter) was used with a blood flow rate from 40 to 100 mL/min and a total extracorporeal blood volume of 100 mL. The session duration and UF rate (100–500 mL/h) were determined by the treating physician. The number of sessions varied between one or two sessions. Single-session UF was performed in twenty patients, while seven patients required double-daily sessions. The mean time of UF treatment was 19 ± 10 h. Intravenous diuretics that were initiated before randomisation were allowed to be continued throughout the duration of treatment. The mean dosage of intravenous furosemide given was 194 ± 175 mg/day.	Intravenous loop diuretics were used according to guideline recommendations under the supervision of an experienced HF cardiologist. Intravenous diuretics that were initiated before randomisation were allowed to be continued throughout the duration of treatment. The mean dosage of intravenous furosemide given was 153 ± 115 mg/day.
Costanzo et al., 2016 (AVOID-HF) [5]	The Aquadex FlexFlow System was used at an initial rate between 150 and 250 cc/h, which was determined by the patient’s initial systolic blood pressure. The therapy was adjusted according to the patient’s response. UF was administered at an average rate of 138 ± 47 mL/h for a mean duration of 80 ± 53 h. Diuretics were withheld for the duration of treatment. Vasoactive drugs were not used, except as a rescue therapy.	Mixed intravenous bolus and infusion of loop diuretics were used according to guidelines and adjusted according to the patient’s response (vital signs and renal function). A mean daily dose of 271.26 ± 263.06 mg of furosemide-equivalent loop diuretic was given. Vasoactive drugs were not used, except as a rescue therapy.
Şeker et al., 2016 [22]	UF with a maximum rate of 500 cc/h. The rate of blood flow was set to 50–100 mL/min. The duration and rate of UF were determined by the clinician. The mean UF duration was 20.5 ± 4.6 h. All forms of intravenous and oral diuretics were withheld for the duration of UF.	Maximum tolerable IV furosemide dose was used as bolus or continuous infusion. The mean daily dose of furosemide given was 164.1 ± 51.3 mg.
Hu et al., 2020 [16]	The FQ-16 type HF ultrafiltration dehydration device was used, with a blood flow rate of 25–40ml/min. The initial UF rate was set to 200–300ml/h, with a mean UF duration of 10.8h/day. The rate and duration of UF were adjusted by the physician depending on the condition of the patient based on blood pressure monitoring.	IV loop diuretics (mean torasemide dose = 20 mg/day) were used along with vasopressin V2 receptor antagonist (mean tolvaptan dose = 10 mg/day).

UF, ultrafiltration; Values after ± denote standard deviations.

**Table 3:** Study outcomes.

First Author	Bart [3]	Bart [10]	Chung [21]	Costanzo [7]	Costanzo [5]	Giglioli [4]	Hanna [6]	Hu [16]	Marenzi [17]	Şeker [22]
Year	2005	2012	2014	2007	2016	2011	2012	2020	2014	2016
Change in sodium level in UF (mmol/L)	−0.5 ± 2.57 (48 h)	−2.3 ± 3.5 (96 h)	-	-	-	-	−4 ± 4.35 * (96 h)	5.11 ± 4.35 * (8 days/EoT)	0 ± 4.35 * (Discharge)	−0.5 ± 5.2 (96 h)
Change in sodium level in diuretics (mmol/L)	0 ± 3.36 (48 h)	0.0 ± 3.6 (96 h)	-	-	-	-	−4 ± 4.65 * (96 h)	1.13 ± 4.65 * (8 days/EoT)	1 ± 4.65 * (Discharge)	0.65 ± 5.7 (96 h)
Change in creatinine in UF (mg/dL)	0.1 ± 0.39 (48 h)	−0.12 ± 0.67 (2 months)	−0.13 ± 0.46 (discharge)	0.05 ± 0.42 (3 months) ^†^	−0.30 ± 0.42 (3 months)	−0.55 ± 0.5075 (36 h) ^‡^	0.6 ± 0.5 (96 h) ^§^	−0.01 ± 0.89 (8 days/EoT) ^||^	0.1 ± 0.42 (12 months) ^†^	−0.36 ± 1.1014 (3 month)
Change in creatinine in diuretics (mg/dL)	0.1 ± 0.33 (48 h)	−0.28 ± 0.61 (2 months)	0.12 ± 0.46 (discharge)	0.08 ± 0.30 (3 months) ^†^	−0.26 ± 0.30 (3 months)	0.07 ± 0.2625 (36 h) ^‡^	0.2 ± 0.49 (96 h) ^§^	−0.02 ± 0.60 (8 days/EoT) ^||^	−0.1 ± 0.30 (12 months) ^†^	0.14 ± 1.1014 (3 month)
Change in BUN in UF (mg/dL)	8 ± 10.0 (48 h)	13.81 ± 23.91 (7 days)	-	-	2.5 ± 6.36 (3 months)	-	-	-	6.1 ± 18.9 (Discharge) ^#^	-
Change in BUN in diuretics (mg/dL)	2 ± 7.8 (48 h)	7.23 ± 20.69 (7 days)	-	-	−3.78 ± 4.19 (3 months)	-	-	-	0 ± 17.5 (Discharge) ^#^	-
Change in BNP in UF (pg/mL)	-	-	-	-	−159.9 ± 678.2 (3 months)	-	-	−1144 ± 1435 (EoT)	−398 ± 527 (Discharge) **	-
Change in BNP in diuretics (pg/mL)	-	-	-	-	−201.3 ± 618.31 (3 months)	-	-	−654.02 ± 889.65 (EoT)	−481 ± 539.26 (Discharge) **	-
Change in NT-proBNP in UF (pg/mL)	-	−814 ± 9239 (96 h)	-	-	-	−3266 ± 6547 (36 h) *	−2291 ± 6547 (48 h) *	-	-	−5567 ± 3855 (96 h)
Change in NT-proBNP in diuretics (pg/mL)	-	−979 ± 2902 (96 h)	-	-	-	−1436 ± 4028.5 (36 h) *	-2547 ± 4028.5 (48 h) *	-	-	−4393 ± 5155 (96 h)

* denotes SD imputed from Bart, 2012 and Şeker, 2016 at 96 h; ^†^ denotes SD imputed from Costanzo, 2016 for change at 3 months; ^‡^ denotes SD imputed from Bart, 2005 and Costanzo, 2016 (24 and 48 h); ^§^ denotes SD imputed from Bart, 2012 and Costanzo, 2016 at 96 h; ^||^ denotes SD imputed from Bart, 2012 at 7 days; denotes imputed SD calculated from *p* value between arms; ^#^ denotes SD imputed from Bart, 2012 and Costanzo, 2016 at discharge; ** denotes SD imputed from Costanzo, 2016 for change at discharge; Values given as mean ± SD.

## Data Availability

The results of our data extraction and synthesis can be found at https://docs.google.com/spreadsheets/d/15RwoXO-h4C7a5Zr0s0cd9f7xKosICl0RIF5N3x5IcOE/edit?usp=sharing (accessed on the 22 January 2023).

## Supplementary Materials

The following supporting information can be downloaded at: https://www.mdpi.com/article/10.3390/jcm12082793/s1, Figure S1: Change in NT-proBNP from baseline against Log10Time; Figure S2: Change in BNP from baseline against Log10Time; Figure S3: Change in Creatinine from baseline against Log10Time; Figure S4: Change in BUN from baseline against Log10Time; Figure S5: Change in Sodium from baseline against Log10Time; Figure S6: Table for the Risk of Bias Assessment for BNP; Figure S7: Summary of the Risk of Bias Assessment for BNP; Figure S8: Table for the Risk of Bias Assessment for Creatinine; Figure S9: Summary of the Risk of Bias Assessment for Creatinine.
