# Supplementary material for: Ultrafiltration versus Diuretics on Prognostic Cardiac and Renal Biomarkers in Acute Decompensated Heart Failure: A Systematic Review and Meta-Analysis
Source: J Clin Med. 2023 Apr 9;12(8):2793. doi: 10.3390/jcm12082793 (PMC10141346; doi:10.3390/jcm12082793)
Supplement: Supplementary file 1 [file jcm-12-02793-s001.zip › jcm-2205882-supplementary.pdf]

Supplementary Materials

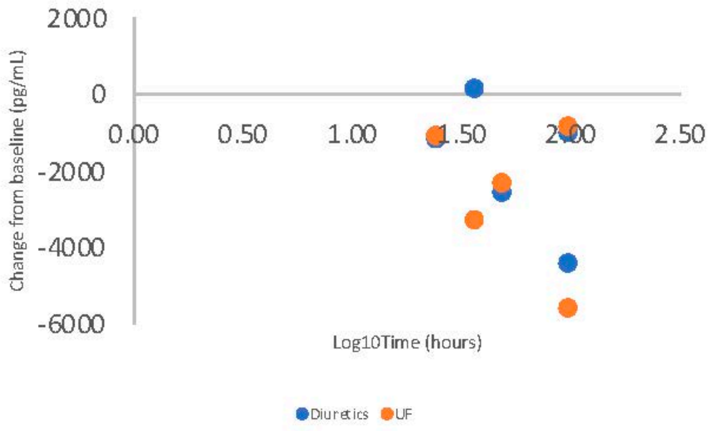

Figure S1. Change in NT-proBNP from baseline against Log10Time.

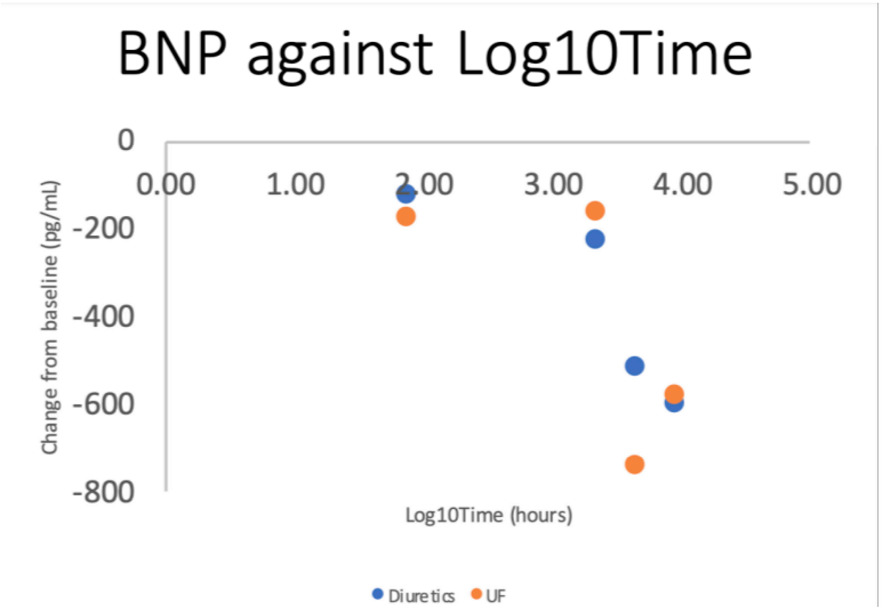

Figure S2. Change in BNP from baseline against Log10Time.

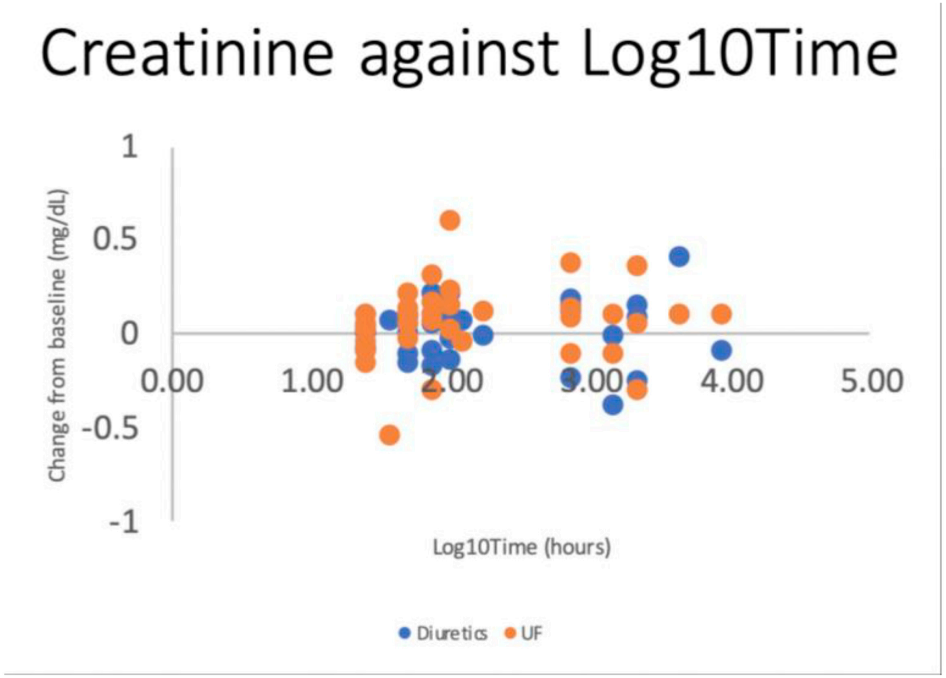

Figure S3. Change in Creatinine from baseline against Log10Time.

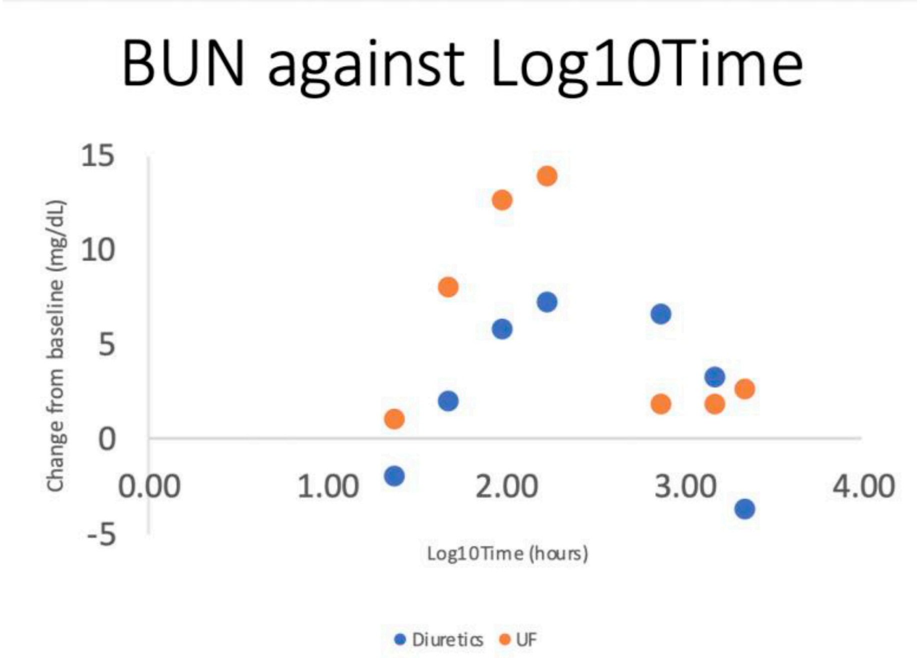

Figure S4. Change in BUN from baseline against Log10Time.

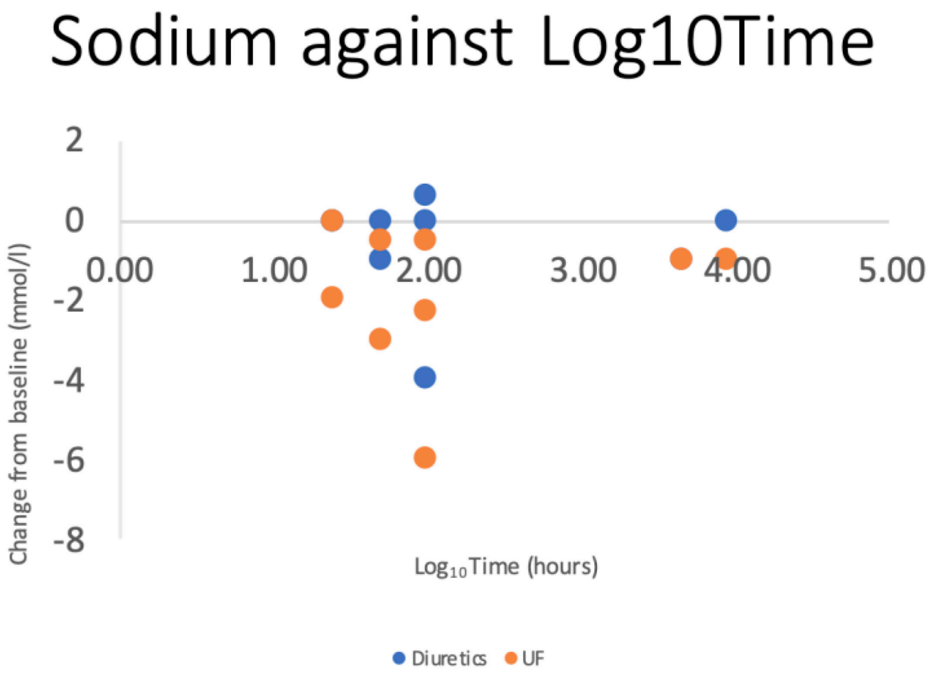

Figure S5. Change in Sodium from baseline against Log10Time.

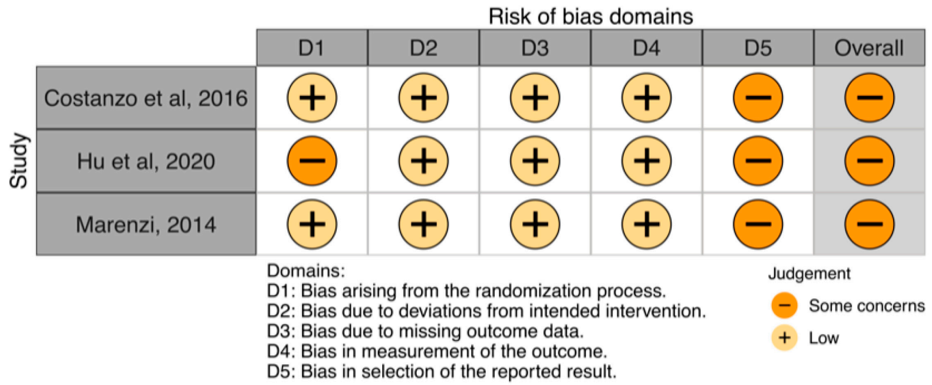

Figure S6. Table for the Risk of Bias Assessment for BNP.

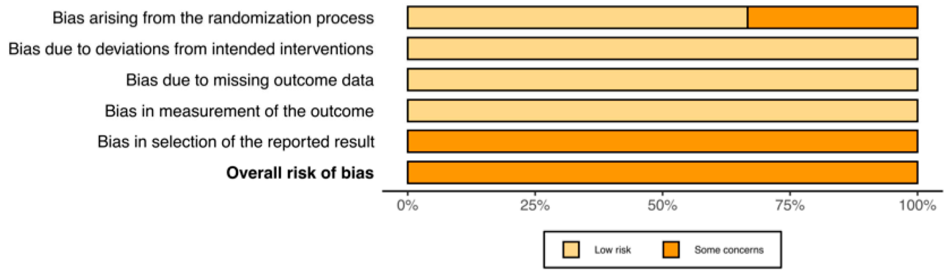

Figure S7. Summary of the Risk of Bias Assessment for BNP.

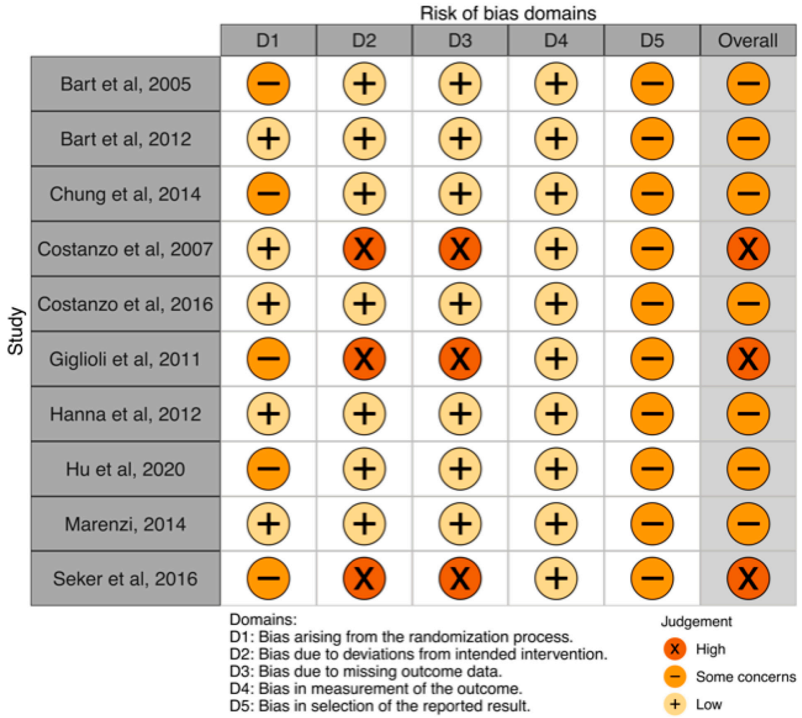

Figure S8. Table for the Risk of Bias Assessment for Creatinine.

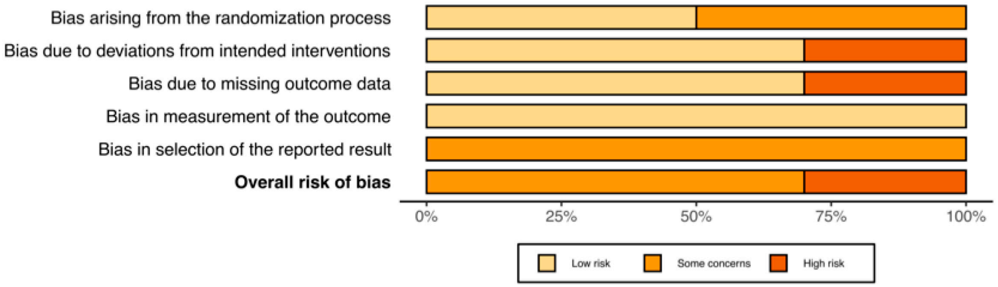

Figure S9. Summary of the Risk of Bias Assessment for Creatinine.
